# A novel hypothesis on the relationship between maternal education and obesity in children; the mediating role of maternal and child self-control—ABIS a population-based cohort study

**DOI:** 10.3389/fpubh.2025.1548949

**Published:** 2025-05-19

**Authors:** Pär Andersson White, Johnny Ludvigsson, Michael P. Jones, Tomas Faresjö

**Affiliations:** ^1^Division of Pediatrics, Department of Biomedical and Clinical Sciences, Linköping University, Linköping, Sweden; ^2^Crown Princess Victoria Children’s Hospital, Linköping University Hospital, Linköping, Sweden; ^3^School of Psychological Sciences, Macquarie University, Sydney, NSW, Australia; ^4^Department of Health, Medicine and Caring Sciences, General Practice, Linköping University, Linköping, Sweden

**Keywords:** socioeconomic status, socioeconomic disparities in health, obesity, self-control, body mass index

## Abstract

**Background:**

Low socioeconomic status, measured by maternal education, increases the risk of obesity in children in high-income countries. This paper presents our hypothesis that self-control mediates the observed association.

**Methods:**

Data from the All Babies in Southeast Sweden (ABIS) cohort, which includes children born 1st Oct 1997–1st Oct 1999 in southeast Sweden with data on BMI, were available for *N* = 5,447 at age 19 out of the original cohort of *N* = 17,055 participants (31.9%). We estimated maternal self-control through behaviors related to self-control, first using a latent variable constructed using the variables breastfeeding duration, maternal smoking during pregnancy, maternal smoking during the first year of the child’s life, and participation with biological samples. In a second model, we also included maternal BMI. Child self-control was measured using the Hyperactivity/Inattention subscale of the Strength and Difficulties Questionnaire (SDQ).

**Results:**

We found that in the relationship between maternal education and BMI/obesity risk of the child at age 19, two indirect paths, maternal self-control and child self-control, mediated 85% of the effect on BMI (model 1) and 87.5% of the effect of obesity risk. Adding maternal BMI (model 2) to the latent maternal self-control variable increased the mediated indirect effect to 95% of the total effect for BMI and 94% of the total obesity risk.

**Conclusion:**

We conclude that maternal self-control and child self-control may mediate most of the effect of low maternal education on BMI/obesity at age 19. The central role of self-control in health inequality, especially for the persistence of health inequalities in the welfare state, may have important implications and should be included when theories of health inequalities are constructed. However, future studies are needed to test the hypothesis described in this paper using additional measures of self-control and executive functions.

## Introduction

Low socioeconomic status (SES), measured by maternal education, increases the risk of obesity in children in high-income countries (1). However, the mechanism through which this and other health inequalities occur has been debated for decades since the publication of the Black Report (2). In this paper, we investigate the role of self-control in body mass index (BMI) and obesity inequality using a Swedish birth cohort. Sweden is an especially appropriate country for this type of study because of the strong welfare state that reduces the effect of other pathways to obesity (3).

**Figure 1:**
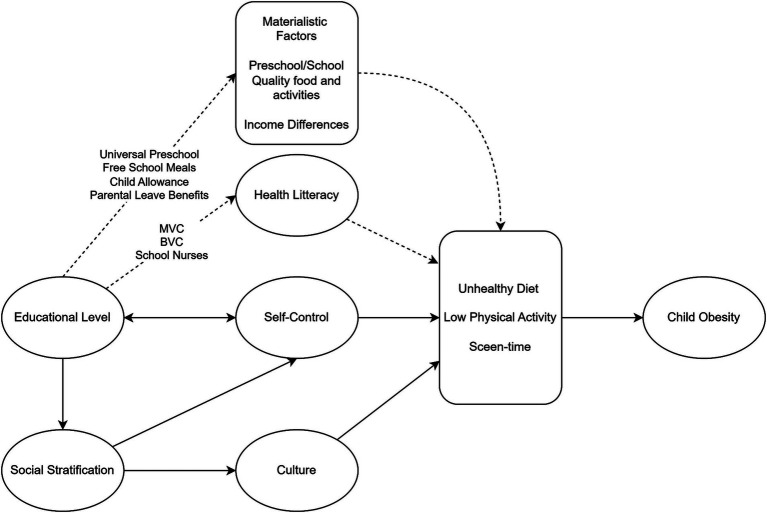
Diagram of pathways between maternal education and child obesity in Sweden. Dashed lines represent pathways interrupted by social and economic policies in Sweden. BVC: Barnavårdcentral = Children’s healthcare centers including dietary recommendation and follow-up of height and weight development; MVC: Mödravårdcentral = Maternal health services including dietary recommendations; School nurses: follows BMI during school years with dietary recommendations.

One prominent theory in health inequality research has been the neo-materialistic theory (3–5). Many materialistic pathways exist in developing high BMI/obesity in high-income countries (see Figure 1). Better quality childcare, preschool, and schools, including fewer children per teacher, better facilities, and higher food quality, will inevitably lead to differences in the child’s environment which may affect BMI and obesity risk. High-income families can pay for such higher quality in countries with a market system for childcare, preschools, and schools. Proponents of the neo-materialistic theory have proposed that this theory better explains the observed differences in health between countries and states with different levels of income inequality than the psychosocial theory (6).

The importance of psychosocial stress for health inequalities has been proposed in the work of Michael Marmot and Richard Wilkinson (7, 8). Their psychosocial theory of inequality states that social hierarchies induce status stress in humans, just like in animals living in social hierarchies. Status stress is thought to increase with higher levels of income inequality and thus explains why health differences are more significant in unequal societies (9). Psychosocial theories have expanded to include psychological theories like Bandura’s self-efficacy theory, where experiences of lack of mastery and resilience lead to low self-efficacy, reducing coping abilities (10). Low-SES individuals are thought to have experienced more negative outcomes, which have led to vicious circles of low self-efficacy and learned helplessness, leaving them vulnerable to the adverse effects of stress (11). Another proposed psychosocial explanation of health inequality is health literacy differences (12). A lack of information and knowledge of health behavior could potentially explain a higher prevalence of obesity in low-SES families in some countries.

The personal characteristics theory, advocated by Johan Mackenbach, suggests that differences in health behaviors due to individual characteristics, such as differences in the Big Five personality traits and IQ, lead to differences in health outcomes (13, 14). This theory is connected to the behavioral theory suggested already in the Black report, where differences in health behaviors are thought to explain health inequalities (3).

Although important for the debate on explanations of health inequality, the behavioral theory failed to explain why behaviors that negatively impact health are more common in low SES families. The personal characteristics theory comes closer to an explanation by recognizing that personal characteristics like psychological traits and cognitive ability explain much of the observed health differences. This theory suggests that the genetic traits that lead to higher SES through upward social mobility are also associated with better health behaviors (14–16).

To get even closer to why behavioral inequality occurs, we must take a closer look at the patterns of health inequality. Such a closer examination reveals that not all health outcomes are more common in low SES groups. Outcomes with a clear connection to the human reward system show an association with low SES, in children obesity due to high calorie diet, in adults liver disease due to alcohol consumption (17), lung cancer due to smoking (18), and cardiovascular disease due to diet/smoking and alcohol use (19). Other outcomes not caused by rewarding behaviors or with unknown etiology show little or no association with SES, e.g., allergies (13), most autoimmune diseases in children (14), and some cancers like prostate cancer in adults (20).

Human behavior and decision-making is a struggle between the reward system, the limbic system, which is aimed at immediate gratification/reward and avoiding immediate negative reinforcers, and our cortex, which predicts what behaviors serve our long-term goals (21). Central to our ability to choose long-term goals instead of immediate reward, to self-control, is the prefrontal cortex (PFC). The PFC needs to control the impulses from the limbic system, both positive and negative reinforcers (22). This PFC control is critical in situations that require behavioral change, such as smoking cessation during pregnancy, where environmental triggers (e.g., stress or boredom) will act as triggers of the behavior (23). Self-control has been defined as the ability for “*Self-initiated regulation of thoughts, feelings, and actions when enduringly valued goals conflict with momentarily more gratifying goals*” (24).

The role of self-control, prominent in other fields of social science, most notably in criminology and the General Theory of Crime (25), has not been emphasized in theories of health inequality. This is puzzling as self-control is more important than IQ for educational attainment (24, 26), and longitudinal evidence shows that self-control predicts educational level (24, 27). Further, behaviors and outcomes of low parental education and low self-control overlap (28, 29). Recent work has found SES-related differences in executive function (30), and MRI and fMRI studies have shown differences in connectivity and thickness of the prefrontal lobe between SES groups (31, 32). The current dominant theory in obesity research, the energy-balance model, proposes the obesity epidemic to an environment rich in highly processed, cheap, palatable foods with a high sugar and fat content that stimulate the brain’s reward system and drive hedonic eating (33). Still, to our knowledge, only one study has investigated the mediating role of self-control in the relationship between early-life SES and obesity. That study found evidence of a mediating effect of an individual’s self-control for adult adiposity and inflammation (34). However, to date, no studies have investigated the role of both parental and child self-control in this relationship.

Our study aims to estimate the mediating effect of maternal and child self-control in the relationship between childhood SES measured by maternal education and the development of higher BMI and obesity in early adulthood.

The difference in group level of self-control between SES groups makes causal interpretation of mediating effects between SES and self-control-related behaviors and outcomes difficult, e.g., one outcome like obesity could be interpreted as mediating another self-control-related behavior like criminality (35). Several potential mediators between parental SES and child obesity have been identified in observational studies; these include smoking during pregnancy and breastfeeding (36). However, the causal relationship between these variables and child obesity has been challenging to establish, and at least one randomized controlled trial (RCT) that successfully increased breastfeeding refuted any effect of breastfeeding on childhood obesity, odds ratio of obesity 1.09 (95% CI, 0.92–1.29) in the intervention group (37). Although it is not impossible that breastfeeding duration and smoking might have biological effects on obesity development, both are also behaviors associated with self-control (38, 39).

We hypothesize that maternal self-control confounds the association between breastfeeding/smoking and child obesity. Based on this hypothesis, we use these self-control-related behaviors as measures of maternal self-control to create a latent variable of this ability. We then analyse the mediating effect of self-control in the maternal education—child obesity relationship.

We hypothesized that maternal self-control would mediate most of the effects of maternal education, but that child self-control would contribute with an additional independent indirect effect.

## Materials and methods

### Study participants

The All Babies in Southeast Sweden (ABIS) study is a prospective population-based cohort that includes children born between October 1st, 1997, and October 1st, 1999, in southeast Sweden. The only exclusion criterion used was that participating parents needed to be able to understand Swedish. Of approximately 21,700 children born during the recruitment period, 17,055 chose to participate (78.6%). Participants were asked to answer an online questionnaire at age 19.57 (SD 0.39) years; 5,447 (31.9% of the original cohort) participated with information on height and weight. The loss-to-follow-up analysis indicated that the participants who were lost to follow-up at age 19 had several notable characteristics. These individuals tended to have mothers with lower levels of education, a higher likelihood of maternal smoking during pregnancy or the child’s first year, and shorter breastfeeding durations. Additionally, they scored higher on the Strengths and Difficulties Questionnaire (SDQ) Hyperactivity scale at age 8, participated in the ABIS study with fewer biological samples, and were more likely to be male. There was no significant difference in maternal BMI at the one-year follow-up (see Supplementary Table S1).

### Study site: Sweden

Sweden is an especially appropriate country for investigating the role of self-control in the overrepresentation of obesity in lower SES groups because of its strong welfare state that reduces the effect of other pathways to obesity (40). In Sweden, social and family policies lessen the importance of materialistic pathways from SES to child obesity through universal access to preschools and schools (including free high-quality meals) and child allowance/parental leave benefits (41). The importance of health literacy is also reduced in Sweden as parents get universal, repeated information on appropriate child nutrition from qualified midwives and nurses who also regularly measure the height and weight of children and inform the parents of the child’s BMI development from the prenatal period to adolescence (42). Recent studies show the effectiveness of the Swedish universal child health care (CHC) program in improving health literacy. It has been shown that 60% of participants from disadvantaged families increase their health literacy after participation in the CHC program and that additional interventions like home visiting programs do not increase this number (43). Since Sweden is a free society where social mobility is theoretically and practically possible, the path from SES to self-control and related health behaviors remains open.

### Outcome

The participants reported their height and weight in an internet-based questionnaire at age 19. Body mass index (BMI) was calculated as BMI = weight (kg)/height (m)^2^. Obesity at age 19 was defined as BMI > 30, in accordance with the World Health Organization’s definition of obesity.

### Exposure

The mothers reported maternal education in the initial questionnaire at the participants’ births. The educational level was classified according to the International Standard Classification of Education (ISCED) and graded in three levels: low maternal education = ISCED level I–II, middle maternal education = ISCED level III–IV, and high maternal education = ISCED level V–VII.

### Mediators

**Figure 2:**
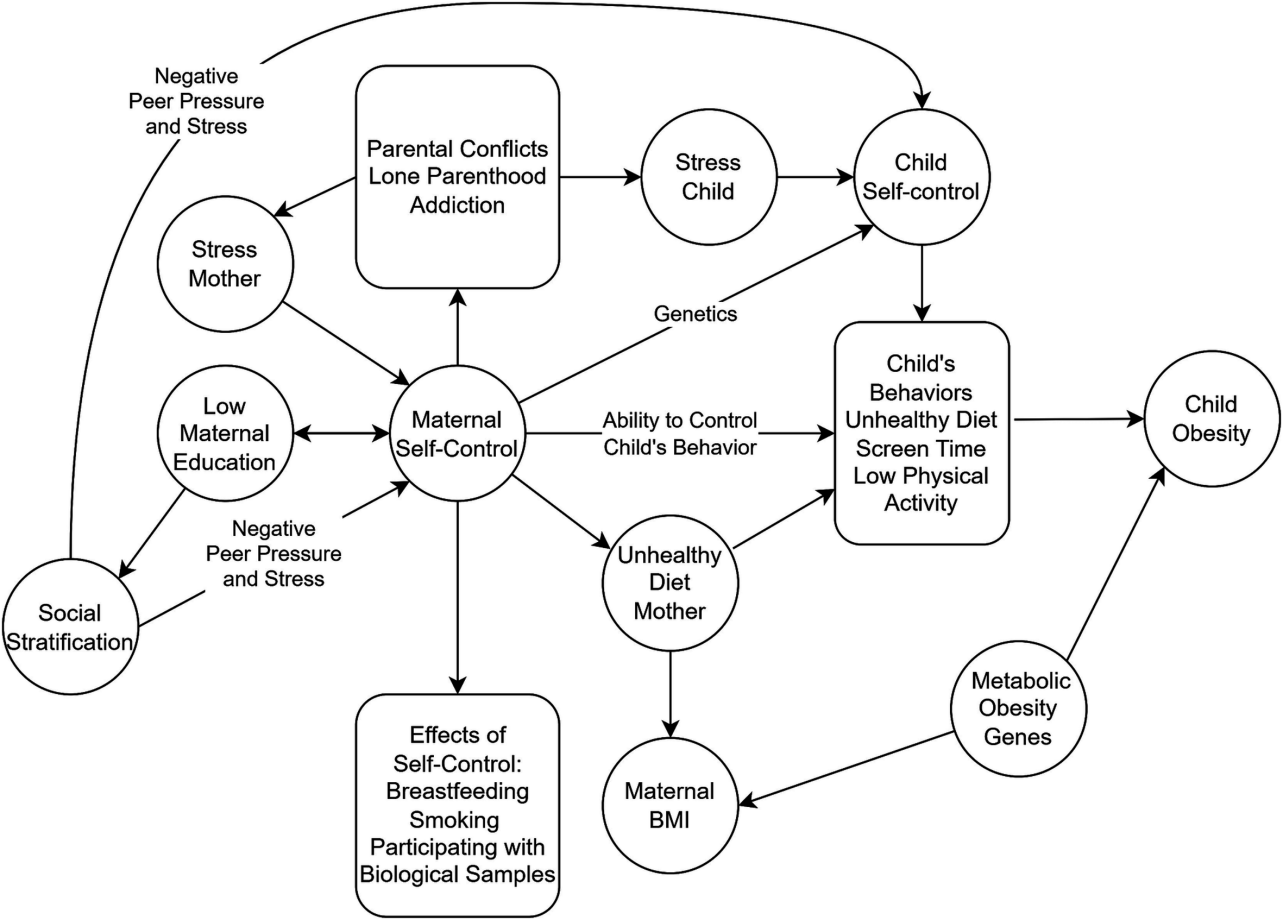
Hypothetic pathways from low maternal education to child obesity. Low maternal education reduces maternal self-control directly and leads to low social stratification that feeds back through risk of negative peer pressure and stress, further reducing self-control. Low maternal self-control decreases the ability to control child’s diet and behaviors and increases risk of unhealthy diet in the mother. Low maternal education increases the risk of other negative self-control-related behaviors like parental conflicts, lone parenthood, and addiction that increases maternal and child stress leading to reduced self-control. Note that self-control-related variables like smoking, breastfeeding, and maternal BMI do not act as mediators on the pathway from maternal education – maternal self-control – child’s behaviors to child obesity.

We measured maternal self-control via a combination of observed behaviors that were available in our dataset and known to be relevant to this construct. These behaviors were combined into a single maternal self-control metric as a latent variable within a structural equation model (SEM). Our measure of self-control is thus constructed just like a latent variable based on a questionnaire where multiple questions about self-control-related behaviors are combined into one measure (44). We used variables that randomized controlled studies indicate do not have a causal relationship with obesity; thus, any mediating effect of the latent variable created will be through association with other pathways related to self-control (see Figure 2).

Variables related to maternal self-control that could be used to construct a latent self-control variable available in the ABIS study included breastfeeding duration, smoking during pregnancy, maternal smoking during the child’s first year of life, and participation in the ABIS study with biological samples. All of these variables represent behaviors that require self-control. The prefrontal cortex needs to overcome the reward system in smoking during pregnancy/a child’s first year (45). In breastfeeding, the PFC needs to control negative reinforcers like overcoming the additional work, including reduced sleep and sometimes pain, compared to bottle-feeding for the potential benefits of the child’s long-term health (45). Studies have shown that mothers with ADHD symptoms have a decreased chance to follow recommendations about 6-month exclusive breastfeeding and attributed this to the fact that the ability to persist in exclusive breastfeeding over time requires attention, perseverance, and focus (46). Participating in a research study with biological samples also involves self-control, as the parent needs to overcome a painful procedure for the child and take the time necessary to collect the samples, all for the long-term benefit of scientific progress (47).

Second, we added maternal BMI (reported when the child was 1 year old) to show maternal self-control’s effect when this behavioral outcome is also included. Maternal BMI is the self-control-related variable that overlaps most with a child’s BMI as self-control will affect the mother’s eating habits, leading to her BMI, and these eating habits will overlap with the child’s. However, maternal BMI and child BMI do not perfectly overlap, as another pathway with a clear SES association in child obesity is the lack of ability to change behavior according to recommendations when overweight and obesity are detected in the child (see Figure 2) (48).

Child self-control was estimated by the hyperactivity/inattention subscale of the Strengths and Difficulties Questionnaire (SDQ-HI); the Swedish version is available at the SDQ homepage (https://www.sdqinfo.org/py/sdqinfo/b3.py?language=Swedish). The SDQ-HI subscale was initially developed to assess self-regulation difficulties, but it shows good face validity for measuring both the presence and absence of self-control (49). The SDQ-HI comprises five questions associated with five components of hyperactivity and inattention: attentional control, “easily distracted, concentration wanders;” inhibitory control, “thinks actions through before acting;” perseverance, “sees tasks through to the end, good attention span,” hyperactive behaviors described as “restless, overactive, cannot stay still for long” and “constantly fidgeting or squirming” which is related to inhibitory control (50). Parents answered whether a sentence presenting the behavior or emotion was not true, somewhat true, or undoubtedly true. The questionnaire was scored with each item giving a score between 0 and 2, resulting in a total score between 0 and 10, with low values indicating high self-control (51). This subscale has been shown to have convergent and discriminant validity with other commonly used measures of childhood self-control, including Children’s Behavior Questionnaire (CBQ) attentional focus and inhibitory control measurements (49).

### Ethics approval and consent to participate

Informed consent to participate was obtained from the parents or legal guardians of any participant under the age of 16. Parents were given oral, written, and video information before providing informed consent to participate in ABIS. In follow-up in adulthood (age >18 years), only the participants themselves were given information and gave their consent to continued participation in the study. The ABIS study was approved by the research committee at Linköping University (Dnr 96-287, Dnr 99-321, Dnr 03-092, Dnr 2013/253-32, Dnr 2016-427-32) and Lund University (LU 83–97) in Sweden.

### Statistical analysis

Relative risks (RR) with 95% Confidence Intervals (CI) were estimated using a generalized linear model with a log link and robust variance estimation with confounders entered as covariates in multivariate regressions (52). RR values represent estimates of the increase in risk per step on the scale of ordinal independent variables (IVs) and comparing categories for binary IVs. The potential role of maternal self-control was analyzed in two separate structural equation models for two outcomes: BMI (quantitative) and obesity (dichotomous). In all models, the association between maternal education and BMI/obesity at 19.57 (SD 0.39) years of age was evaluated through SEMs since they can decompose the total association between maternal education and child obesity into that which operates directly between these two variables and that which operates indirectly via (i) maternal and (ii) child self-control. Maternal education was the primary IV, BMI/obesity was the dependent variable (DV), and maternal self-control and child self-control were included as intermediate variables between IV and DV. Maternal self-control is represented in the model as a latent variable, as described above. Both estimates of parameters representing the direct association between IV and DV, as well as the indirect association via maternal self-control and child self-control, were calculated. The indirect association is reported along with standard errors and expressed as a percentage of the total association. We view this percentage as the most directly relevant since it quantifies how much of the association between IV and DV is indirect via measures of self-control and therefore is potentially mediated by self-control. The indirect association is reported along with standard errors and expressed as a fraction of the total association. Formal statistical inference was made using the nonparametric bootstrap and 1,000 bootstrap samples due to violation of the assumption of multivariate normality. All parameter estimates (path coefficients) are reported in a standardized form. The fit of the structural equation models is not as relevant in this instance since our hypotheses do not relate to the model as a whole, but to specific parameters within the model. However, the root mean square error of approximation (RMSEA) is reported as an overall measure of model fit since it quantifies the discrepancy between implied and observed correlation matrices and is independent of sample size.

## Results

The mean BMI at age 19 was 23.2 (SD 4.15), and the prevalence of obesity was 5.2% (see Table 1). Average BMI increased with lower maternal education (see Table 2). Children with low maternal education had a 66% higher risk of developing obesity at age 19. The prevalence in the middle educational level was 5.9%, and the relative risk (RR) of obesity was 1.46 (CI 1.13, 1.88). For low maternal education and obesity, the prevalence was 6.7%, yielding an RR of 1.66 (1.07, 2.57).

**Table 1:** Characteristics of the population at follow-up 19.57 (SD 0.39) year of age, *n* = 5,447 (33.3% of the original cohort).

Characteristics	*N*	%	Mean	SD
Maternal education				
High	2065	37.9		
Middle	2,927	53.7		
Low	356	6.5		
Missing	100	1.8		
Ethnicity mother				
Swedish	5,050	92.7		
Other	309	5.7		
Missing	88	1.6		
Child’s sex				
Male	2,471	45.4		
Female	2,976	54.6		
missing	0	0		
SDQ hyperactivity				
Score at 8 yrs.	1781	32.7	2.10	2.01
Missing	3,666	67.3		
Biological samples				
Sample data	5,447	100	3.19	2.11
Missing	0			
Smoking pregnancy				
Yes	509	9.3		
No	4,835	88.8		
Missing	103	1.9		
Smoking^a^				
Yes	168	3.1		
No	3,752	68.9		
Missing	1,527	28.0		
Breastfeeding				
1 month	316	5.8		
2–4 months	1,612	29.6		
5–6 months	1,400	25.7		
>7 months	357	6.6		
Missing	1762	32.3		
Maternal BMI^a^				
BMI	3,742	68.7	23.73	3.82
Missing	1705	31.3		
BMI child age 19				
BMI	5,447	100	23.24	4.15
Missing	0	0		
Overweight	1,260	23.1		
Obese	285	5.2		

a Maternal smoking status and maternal BMI were measured in the child aged 1-year follow-up.

BMI, body mass index; SD, standard deviation; SDQ, strength and difficulties questionnaire.

**Table 2:** Maternal educational level and mean BMI and relative risk of overweight and obesity in child at age 19 years.

Educational level	BMI		Obesity			
	Mean	SD	*N*	%	RR	CI
High education	22.94	3.65	84	4.1	1.00	
Middle education	23.41	4.43	174	5.9	1.46	(1.13, 1.88)
Low education	23.50	4.56	24	6.7	1.66	(1.07, 2.57)

BMI, body mass index; CI, confidence intervals; RR, relative risk.

### Mediation analysis

**Figure 3:**
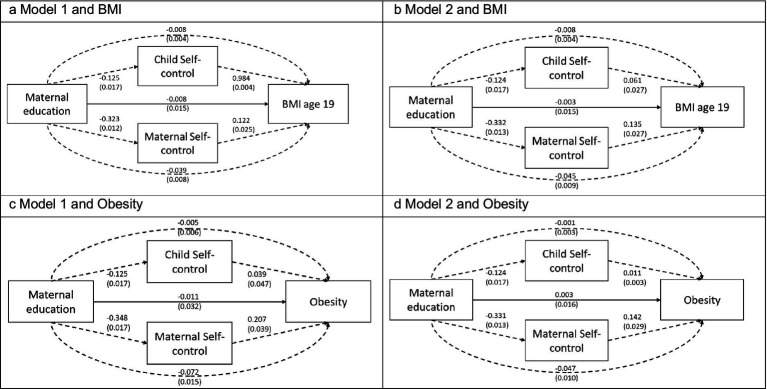
Direct and indirect pathways between maternal education and child BMI/obesity at age 19. **(a)** Shows the results from model 1 (maternal self-control is a latent variable constructed using four variables: smoking during pregnancy, smoking during the child’s first year of life, breastfeeding duration, and participation with biological samples) with BMI as an outcome. **(b)** Shows the result of model 2 (adding maternal BMI to the maternal self-control variable) with BMI as an outcome. **(c)** Shows the results of model 1 and **(d)** results of model 2 with obesity as an outcome.

In Model 1, the latent variable for maternal self-control (constructed using four variables: smoking during pregnancy, smoking during the first year of the child’s life, breastfeeding duration, and participation with biological samples in ABIS) accounted for −0.039 (SE 0.008, *p* = <0.001) out of a total effect of −0.055 (SE 0.014) representing 70.91% of the total effect. The second indirect path through child self-control accounted for −0.008, representing 14.55% of the total effect. The remaining direct effect was estimated to be −0.008 (SE 0.004, *p* = 0.034) or 14.55% of the total effect (see Table 3; Figure 3). Thus, most of the effect of low maternal education on the risk of obesity development in the child was mediated by the latent maternal self-control variable.

**Table 3:** Mediation model of the relationship between maternal education and BMI at age 19.

	Model 1^a^				Model 2^b^			
	Estimate	S.E.	*p*-value	% Mediated	Estimate	S.E.	*p*-value	% Mediated
Total effect maternal education	−0.055	0.014	<0.001		−0.056	0.014	<0.001	
Indirect effect child self-control	−0.008	0.004	0.034	14.55%	−0.008	0.004	0.036	14.29%
Indirect effect maternal self-control	−0.039	0.008	<0.001	70.91%	−0.045	0.009	<0.001	80.36%
Total indirect effect	−0.047	0.009	<0.001	85.45%	−0.053	0.010	<0.001	94.64%
Total direct effect	−0.008	0.015	0.570	14.55%	−0.003	0.015	0.842	5.36%

The model fit is reported as RMSEA (root mean square error of approximation) with 90 percent confidence intervals, a RMSEA below 0.05 indicates good model fit. The RMSEA for model 1 was 0.020 (0.016, 0.024) for model 2: 0.040 (0.037, 0.043).

a Maternal self-control is estimated using smoking during pregnancy, smoking during the child’s first year of life, total breastfeeding duration, and participation with biological samples.

b Maternal self-control was estimated using variables in model 1 and maternal BMI in the ABIS 1-year follow-up.

Results using a dichotomous outcome variable for obesity showed similar results (see Table 4). Maternal self-control accounted for −0.072 (SE 0.015, *p* < 0.001) out of a total effect of −0.088 (SE 0.028), representing 81.82% of the total effect. The indirect path through child self-control accounted for −0.005 (SE 0.006, *p* = 0.421), representing 5.68% of the total effect (n.s.). The remaining direct effect was −0.011 (SE 0.032, *p* = 0.741), representing 12.5% of the total effect (n.s.).

**Table 4:** Mediation model of the relationship between maternal education and obesity at age 19.

	Model 1^a^				Model 2^b^			
	Estimate	S.E.	*p*-value	% Mediated	Estimate	S.E.	*p*-value	% Mediated
Total effect maternal education	−0.088	0.028	0.002		−0.045	0.014	0.001	
Indirect effect child self-control	−0.005	0.006	0.421	5.68%	−0.001	0.003	0.631	1.96%
Indirect effect maternal self-control	−0.072	0.015	<0.001	81.82%	−0.047	0.010	<0.001	92.16%
Total indirect effect	−0.077	0.016	<0.001	87.50%	−0.048	0.014	<0.001	94.12%
Total direct effect	−0.011	0.032	0.741	12.50%	0.003	0.016	0.836	5.88%

The model fit is reported as RMSEA (root mean square error of approximation) with 90 percent confidence intervals, a RMSEA below 0.05 indicates good model fit. RMSEA (root mean square error of approximation) for model 1: 0.023 (0.019, 0.027) for model 2: 0.034 (0.031, 0.037).

a Maternal self-control is estimated using smoking during pregnancy, smoking during the child’s first year of life, total breastfeeding duration, and participation with biological samples.

b Maternal self-control was estimated using variables in model 1 and maternal BMI in the ABIS 1-year follow-up.

In Model 2, the latent variable for maternal self-control (constructed using five variables: smoking during pregnancy, smoking during the first year of the child’s life, breastfeeding duration, participation with biological samples, and maternal BMI reported in the ABIS one-year follow-up) accounted for −0.045 (SE 0.009, *p* < 0.001) out of the total effect of −0.056 (SE 0.014) corresponding to 80.36%. The accounted-for effect of the second pathway through child self-control remained unchanged at −0.008 (SE 0.004, *p* = 0.036). The combined indirect paths corresponded to 94.64% of the total effect 0.056.

In the SEM using obesity as an outcome, maternal self-control accounted for −0.047 (0.010, *p* < 0.001) out of a total effect of 0.051, representing 92.16% of the effect. Child self-control accounted for −0.001 (SE 0.003, *p* = 0.631), representing 1.96% of the relationship (n.s.). The direct effect was 0.003 (SE 0.016, *p* = 0.836). Note that the direct effect was positive in this model, indicating that the relationship between maternal education and obesity could potentially have been reversed without the indirect paths. However, this result was not statistically significant.

## Discussion

We present our hypothesis about the pathways between low maternal education and social inequality in early adulthood BMI and obesity. The results confirm our hypothesis that the latent variable we constructed as a measurement of maternal self-control mediates most of the effect of maternal education on a child’s BMI and risk of obesity in early adulthood. Another independent pathway through the child’s self-control was also identified. Together, the two indirect paths mediated 85% of the effect on BMI (model 1) and 87.5% of the effect on obesity risk. With a prevalence difference of 2.6% between high and low maternal education and a 66% higher relative risk for the low educational group, these large mediating effects are highly significant.

BMI differences and obesity are among the earliest diseases to develop during life, with a clear SES gradient in high-income countries (1). That differences in self-control, the way we have defined this parameter, mediates such a large percentage of the effect of maternal education indicates a central role of the pathway from maternal education through self-control (which could be viewed as a personal characteristic) to behavior (diet, physical activity) in the relationship between SES and increased BMI and obesity. It also explains the persistence of inequality in obesity and its many complications, including Type 2 Diabetes, liver disease, and cardiovascular disease in the welfare state (13).

### Determinants of self-control ability

Gottfredson and Hirschi made progress in studying another self-control-related behavior, crime, when they developed their General Theory of Crime (GTC), also called the self-control theory (25). However, in their eagerness to state the importance of their findings, they were quite polemic with previous sociological research. The GTC stated that self-control was genetic or trained at a very early age and that factors during childhood and adolescence that traditionally had been viewed as causes of criminality (like adversities during childhood and peer pressure) were irrelevant (25).

Modern self-control research and neuroscience suggest otherwise (53). Several factors determine self-control differences. These factors include: Genes, training/neuroplasticity, stress, peer pressure, and self-efficacy. Estimates of the heritability of self-control range widely. However, one meta-analysis of twin studies reported a genetic component of 60% (54). ADHD, the current diagnosis for individuals with the lowest control of impulsivity, is partly genetic (55). The prefrontal cortex is not fully developed until the mid-twenties, but neuroplasticity and self-control training can improve it even after that age (56). A role of psychosocial stress in health inequalities suggested by the psychosocial theory of health inequality could be consistent with the central role of self-control in health inequality, as self-control is reduced by stress with activation of the HPA axis and the release of glucocorticoids that reduce prefrontal lobe inhibition of the limbic system (7, 8, 57). Psychosocial stress and its complications, such as reduced sleep, also decrease PFC control (58). Studies have shown that peer pressure affects self-control, and peer interaction can improve self-control and reduce it depending on the quality of the interaction. The presence of peers and acceptance of peers improve self-control, while rejection by peers reduces self-control (59). Peer contagion, the spread of negative behavior between peers, has been shown to increase aggression, bullying, disordered eating, drug use, and depression (60). Having friends with low-risk behaviors and other protective behaviors has been shown to mediate a reduced risk of violence and risk behaviors in adolescents (61). Given the gradient of self-control across SES, high SES individuals will be more likely to experience positive peer pressure to improve self-control. In contrast, low SES individuals will have a higher risk of exposure to negative peer pressure. Self-efficacy also affects self-control; negative experiences of inability to change a behavior or to be successful in a challenging task will influence future self-efficacy in similar situations, i.e., one’s perceived ability to achieve a goal successfully (62). Self-efficacy is essential for the strength of the higher-order goal in a self-control situation (21). Thus, repeated inability to change behavior and outcomes, e.g., weight reduction in obesity, may lead to low self-efficacy. Related to low self-efficacy is the state of learned hopelessness, i.e., the expectation that even if I try, I will not succeed, and the result will be negative. Also related is the state of helplessness, i.e., what I do does not affect the outcome (LH) (11). In LH, people perceive their ability to change the current situation as low and become passive. LH reduces self-control by reducing commitment to the higher-order goal (21, 63).

The difference between parental educational level/SES and other variables related to self-control is the feedback loop by which educational level leads to social stratification that determines a person’s social environment, including the SES and self-control of peers (64). This is not the case for other variables related to self-control, such as breastfeeding duration, physical activity, healthy diet, participation in research, vaccination participation, etc., that are not a source of social stratification and, thus, do not influence who will become an individual’s closest peers.

### Strengths and limitations of the study

The benefit of using outcomes of self-control to measure its role as a mediator is that unlike tests of executive function like the Strobe test or fMRI measurements of PFC activation, our self-control variable is a result not only of the internal reasons for self-control (genes and training/neuroplasticity) but also the external factors (stress, peer pressure, self-efficacy) and culture.

Our indicators and the variables used to measure maternal self-control are, at this stage, not yet validated measurements but tentative variables based on relevant factors available in our data collection. Although we believe this construct shows good face validity, we acknowledge that it relies on assumptions about the relationship between variables that we cannot confirm within the context of the data of our observational study. As described in Figure 2, the relationship between self-control and educational level is bidirectional, and some authors suggest that the effect of low self-control on behavior is mediated mainly by the social effects of a lower educational level, consistent with the social causation explanations (65). We do not argue that this may be part of the explanation, and the relative importance of self-control ability and its downstream social consequences must be investigated in future studies. Future research is required to strengthen the self-control hypothesis, and research should include multiple measurements of parental and child self-control, e.g., the Brief Self-control Scale (66), to compare the results of these questionnaire-based measures with latent variables constructed using behaviors. We are aware that some factors we include in our definition of self-control may have importance for the development of obesity via mechanisms other than self-control, and this is especially true for maternal BMI, where genetics plays an important role.

The lack of other measurements of executive function and imaging data also limited our study. Because of this, we could not separate the effect mediated by internal self-control ability (executive function) from that mediated by external factors. Future research could try to separate these factors by using objective measures like Stroop tests or fMRI activation and compare their effects with constructs of real-life self-control ability, like the latent variable used in this study.

The use of effects of self-control to estimate a latent variable could overestimate its effect if variables related to self-control used in the calculation also have a direct effect independent of self-control on the outcome of BMI/obesity. We argue that the risk of a significant direct effect of the behaviors used in this study is relatively low. The relationship between breastfeeding and a reduced risk of child obesity has been reported in numerous observational studies. Still, in a randomized controlled trial (RCT), where breastfeeding was promoted in randomly selected hospitals, breastfeeding was associated with a slight increase in BMI and not a reduction (37). Another repeated observation in child obesity studies is the increased risk of obesity in children of mothers who smoke during pregnancy (67). Because of ethical reasons, there will never be an RCT on the relationship between smoking during pregnancy and child obesity. However, the only established effect of smoking during pregnancy, supported by animal studies and human epidemiological data, is children being born smaller than expected for their gestational age (SGA) if exposed to smoking during pregnancy (68). Some studies have suggested that SGA could lead to an increased risk of later development of obesity. However, recent studies have shown that this finding was due to methodological issues and that the actual effect of SGA is a reduction of BMI and not an increase in children (69). The impact of passive smoking during childhood is also likely to be neutral or a reduction of BMI, as the effect of cigarette smoke/nicotine is to increase metabolic rate and reduce appetite (70).

There was a significant loss to follow-up in the ABIS study between the inclusion questionnaire, the 8-year questionnaire used to measure SDQ in the child, and the last follow-up at 19 years of age. This missing data could affect the generalizability of our findings. However, previous research has shown that this loss to follow-up in longitudinal studies does not seem to impact the qualitative conclusions of studies on SES inequality (71). Thus, although the exact effect-sizes and levels of inequality might differ from the general population the conclusions on inequality and a mediating effect of self-control should be valid.

Using self-reported height and weight may affect the obesity prevalence observed in our study. Previous research has shown a good correlation between self-reported and measured height and weight. However, many studies indicate that Body Mass Index (BMI) is often slightly underestimated. Men tend to overestimate their height, while women typically underestimate their weight (72). Consequently, both the BMI values and the prevalence of obesity reported in our study may be slightly underestimated.

The pathway between SES, self-control, parental BMI, and child obesity is more complex than the other variables in Model 1. High parental BMI increases the risk of child obesity through genetic factors that regulate metabolism (73). This does not confound our analysis if our assumption is correct that metabolic genetic factors do not affect maternal education (see Figure 2). To our knowledge, no studies indicate a relationship between obesity-related metabolic genes and educational outcomes. However, some studies suggest that obesity could affect education indirectly by reducing self-esteem and increasing the risk of depression (74). Thus, it could be hypothesized that metabolic genes that predispose to weight gain, independent of self-control related behaviors, could reduce educational attainment. To investigate this, a study comparing the educational attainment of people with monogenic obesity (conditions with mutations in the satiety signaling in the hypothalamus that do not involve other brain areas) compared to the general population would be of value. At this time, we believe most of the evidence suggests the relationship between obesity and educational attainment to be mediated by self-control and related dietary behaviors. Still, it cannot be excluded that the very high mediating effect (94.64% for BMI and 94.12% for obesity) seen when maternal BMI is introduced into our mediation model is partly due to the effect of confounding by metabolic genes.

The high percentage of indirect effects mediated by the self-control variables found in this study must be viewed in the context of the study, a Nordic welfare state. A similar study conducted in a high-income country with fewer policies directed at reducing materialistic differences and inequalities (i.e., the USA) would likely have had a more significant unexplained/direct effect on the total effect due to other pathways between SES and BMI related to materialistic differences.

### Implications of the SES – self-control mediating pathway for healthy inequality research

During the first decades of research on health inequalities after the Black Report, research on personal characteristics as an explanation of health inequality was hampered by the fear that it would lead to the victims of inequality being blamed for their situation, which would reduce incentives for implementing structural policies to reduce disparities (14). Although well meant, this view impeded the development of a complete theory of health inequality and the development of policies that counteract one of the core reasons for health inequality: differences in self-control between SES groups.

Theories developed that ignored self-control included materialistic theories like the diffusion of innovation and the fundamental cause theories. According to the diffusion of innovation theory, innovations tend to reach the better-off first before trickling down to the lower classes (75). Today, however, the benefits of innovation rapidly spread in high-income countries, especially health innovations in countries with strong welfare states (e.g., new medications and insulin pumps for type 1 diabetes patients). New evidence of harmful effects of behaviors is not as easily integrated and leads to SES differences. The fundamental cause theory explains this by stating that SES represents a fundamental cause that will affect the possibility of changing behavior when new information on preventing disease emerges (76). The authors of this theory stated that high SES gives “access to resources that can be used to avoid risks or to minimize the consequences of disease once it occurs. We define resources broadly to include money, knowledge, power, prestige, and the kinds of interpersonal resources embodied in the concepts of social support and social networks.” Thus, this theory predicted that SES differences will occur if SES differences in these resources continued. The theory also predicts that policies like “minimum wage, housing for homeless people, capital-gains taxes, parenting leave” would be more effective at addressing these differences than policies directed directly at the health behaviors.

In this paper, we propose a different, although not mutually exclusive, explanation for health inequality: the central role of self-control. Including self-control in a “General theory of health inequality” would allow for understanding the persistence of health inequality in the welfare state and explain why addressing the causes suggested by the fundamental cause theory does not lead to complete equity in health.

### Policy implications of the SES – self-control mediating pathway

A recognition of the central role of self-control for health inequalities should not be misinterpreted in a deterministic way, that health inequalities are inevitable and unmendable to interventions. The ethical imperative to reduce disparities (77) remains unchanged by recognizing that the ability to self-control increases with higher SES. However, understanding that self-control is the central path to social inequalities in the welfare states can lead to a better understanding of what policies are necessary to reduce these inequalities.

A clear understanding of the role of self-control in social health inequalities clarifies how these inequalities should be reduced and gives a rationale for some of the public health policies already in place. Policies likely to reduce health inequalities, including BMI differences/obesity related to differences in self-control, should bypass parental self-control; for example, by providing universal free preschool, school, and after-school activities, including healthy universal free preschool/school meals. Policies should reduce exposure to stimuli with adverse effects on health, e.g., ban advertisements to children, limit access to candy and unhealthy snacks in schools, and set an age limit on energy drinks, alcohol, tobacco, vaping, etc. Policies should also avoid reducing self-control; evidence shows that screen time and social media use reduce self-control (61, 62). Limitations on screen time and social media use could reduce the negative impact of these behaviors on self-control. Further, implementing policies to train self-control could be valuable, e.g., by giving every child attention in preschool/school by teachers in classes with a high teacher-to-student ratio. The Carolina Abecedarian Project found that intervening in early childhood to improve the education of low SES children can improve downstream outcomes related to self-control in adult life, both the educational attainment of the children and their adulthood health outcomes, including obesity, blood pressure, and other metabolic variables (78). Lastly, policies should reduce segregation. Reduced segregation would equalize positive peer pressure and reduce negative peer pressure. The moving-to-opportunity study showed that moving from a neighborhood with low SES to a higher SES neighborhood decreased anxiety symptoms in both parents and children (79); later follow-ups have also shown a reduced risk of obesity and Diabetes in adulthood (80). These studies indicate the critical role of reducing segregation to improve self-control-related outcomes.

## Conclusion

In this study we found that maternal self-control mediated most of the effect of low maternal education on BMI/obesity in early adulthood. Another mediating path through child self-control was also detected. The central role of self-control in health inequality, especially for the persistence of health inequalities in the welfare state, has important public health implications and should be included when theories of health inequalities are further developed. However, future studies are needed to test the hypothesis described in this paper using additional and validated measures of self-control and executive functions.

## Data Availability

The raw data supporting the conclusions of this article will be made available by the authors, without undue reservation.
