# Supplementary material for: A novel hypothesis on the relationship between maternal education and obesity in children; the mediating role of maternal and child self-control—ABIS a population-based cohort study
Source: Front Public Health. 2025 May 19;13:1548949. doi: 10.3389/fpubh.2025.1548949 (PMC12127286; doi:10.3389/fpubh.2025.1548949)

Supplementary material

| **Table S1: Attrition analysis – characteristics of young adults participating in the ABIS study at age 19 year follow-up, compared to participants who had dropped out of the cohort** | | | | | |
| --- | --- | --- | --- | --- | --- |
|  | **Participants in the 19 year follow-up** | | **Drop-outs** | | **P-value** |
| **N participants (%)** | 5447 | (33.3) | 10918 | (66.7%) |  |
| Maternal education |  |  |  |  | <0.001 |
| High | 2065 | (38.6) | 2997 | (28.3) |  |
| Medium | 2927 | (54.7) | 6582 | (62.1) |  |
| Low | 356 | (6.7) | 1018 | (9.6) |  |
| BMI Mother, mean (SD) | 23.73 | (3.82) | 23,74 | (3.95) | 0.905 |
| SDQ Hyperactivity scale, mean (SD) | 2.10 | (2,01) | 2,35 | (2.18) | <0.001 |
| Biological samples, mean (SD) | 3.19 | (2.11) | 2,60 | (2.01) | <0.001 |
| Breastfeeding |  |  |  |  | 0.16 |
| 0-1 Month, N (%) | 316 | (8.6) | 681 | (10.3) |  |
| 2-4 Months, N (%) | 1612 | (43.7) | 2782 | 42.1 |  |
| 5-6 Months, N (%) | 1400 | (38.0) | 2460 | 37.2 |  |
| 7 Months or more, N (%) | 357 | (10.4) | 686 | (9.7) |  |
| Smoking during pregnancy, N (%) | 509 | (9.5) | 1267 | (11.9) | <0,001 |
| Smoking child’s 1^st^ year, N (%) | 168 | (4.8) | 458 | (6.5) | <0.001 |
| Male sex, N (%) | 2471 | (45.4) | 6000 | (55.1) | <0.001 |

Figure S1a. Model 1: Self-control and BMI at age 19. Coefficients of the variables used to construct the latent variable for maternal self-control (smokepr = smoking during pregnancy, smoke1yr = smoking during the child’s first year of life, durnbf = breastfeeding duration, and bloodnr = number of times the participant had participated with biological samples.

Figure S1b. Model 2: Self-control and BMI at age 19. Coefficients for included variables in the latent variable of maternal Self-control (smokepr = smoking during pregnancy, smoke1yr = smoking during the child’s first year of life, durnbf = breastfeeding duration, bloodnr = number of times the participant had participated with biological samples, and bmim_1 = maternal BMI at child age 1 year)

Figure S2. Flow-chart showing loss-to-follow-up at different waves of the ABIS study.


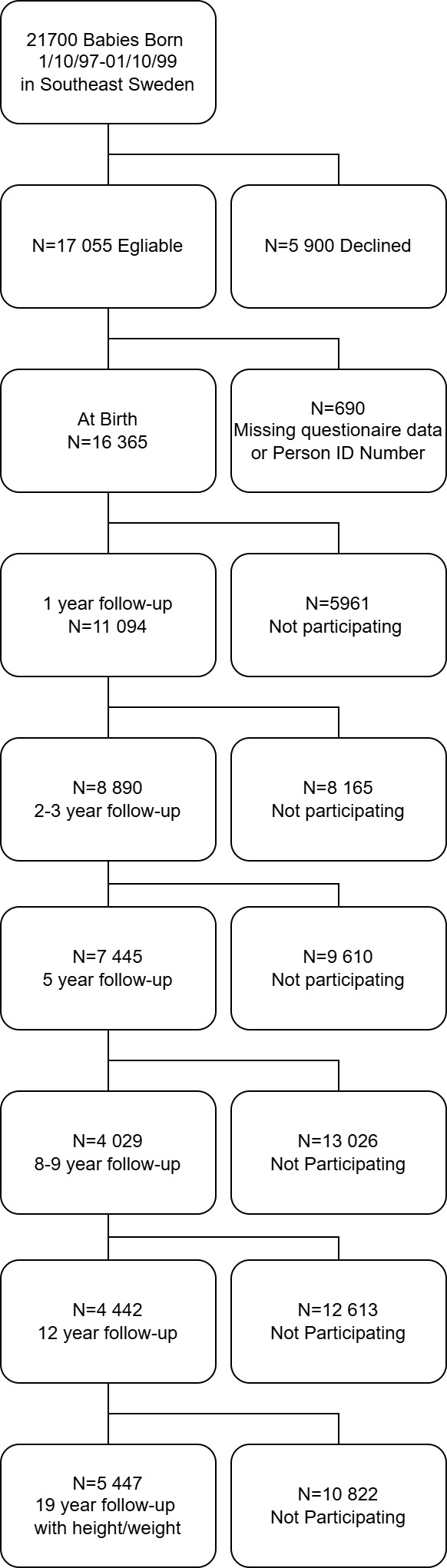

Supplement: Supplementary file 1 [file Table_1.DOCX]
